# Integrative Analyses of Circulating Small RNAs and Kidney Graft Transcriptome in Transplant Glomerulopathy

**DOI:** 10.3390/ijms22126218

**Published:** 2021-06-09

**Authors:** Canan Kuscu, Manjari Kiran, Akram Mohammed, Cem Kuscu, Sarthak Satpathy, Aaron Wolen, Elissa Bardhi, Amandeep Bajwa, James D. Eason, Daniel Maluf, Valeria Mas, Enver Akalin

**Affiliations:** 1 Transplant Research Institute, James D. Eason Transplant Institute, Department of Surgery, School of Medicine, University of Tennessee Health Science Center, Memphis, TN 38163, USA; ckuscu1@uthsc.edu (C.K.); aaron@wolen.com (A.W.); abajwa@uthsc.edu (A.B.); jeason1@uthsc.edu (J.D.E.); 2 Department of Systems and Computational Biology, School of Life Sciences, University of Hyderabad, Hyderabad 500046, India; manjari.hcu@uohyd.ac.in (M.K.); 16ilmb15@uohyd.ac.in (S.S.); 3 Center for Biomedical Informatics, University of Tennessee Health Science Center, Memphis, TN 38163, USA; amoham18@uthsc.edu; 4 Department of Surgery, School of Medicine, University of Maryland, Baltimore, MD 21201, USA; ebardhi@som.umaryland.edu (E.B.); DMaluf@som.umaryland.edu (D.M.); VMas@som.umaryland.edu (V.M.); 5 Montefiore Medical Center, Abdominal Transplant Program, Albert Einstein College of Medicine, Bronx, NY 10467, USA; eakalin@montefiore.org

**Keywords:** transplant glomerulopathy, circulating small non-coding RNAs, miRNAs, tRNA fragments, non-invasive biomarker, RNA-seq

## Abstract

Transplant glomerulopathy develops through multiple mechanisms, including donor-specific antibodies, T cells and innate immunity. This study investigates circulating small RNA profiles in serum samples of kidney transplant recipients with biopsy-proven transplant glomerulopathy. Among total small RNA population, miRNAs were the most abundant species in the serum of kidney transplant patients. In addition, fragments arising from mature tRNA and rRNA were detected. Most of the tRNA fragments were generated from 5′ ends of mature tRNA and mainly from two parental tRNAs: tRNA-Gly and tRNA-Glu. Moreover, transplant patients with transplant glomerulopathy displayed a novel tRNA fragments signature. Gene expression analysis from allograft tissues demonstrated changes in canonical pathways related to immune activation such as iCos-iCosL signaling pathway in T helper cells, Th1 and Th2 activation pathway, and dendritic cell maturation. mRNA targets of down-regulated miRNAs such as miR-1224-5p, miR-4508, miR-320, miR-378a from serum were globally upregulated in tissue. Integration of serum miRNA profiles with tissue gene expression showed that changes in serum miRNAs support the role of T-cell mediated mechanisms in ongoing allograft injury.

## 1. Introduction

Transplant glomerulopathy (TG) is a specific lesion seen in kidney transplant biopsies that is visually characterized by capillary wall widening and double contours. TG is described in the Banff classification as a manifestation of chronic antibody-mediated rejection (ABMR) if peritubular capillary C4d staining is positive and/or microvascular inflammation (MVI) score (glomerulitis and peritubular capillaritis) is ≥2 [1,2]. However, TG has also been described in the absence of DSA or positive C4d or MVI [3,4,5]. Increased chemokines (CXCL9 and CXCL10), chemokine receptor (CXCR3) and inducible costimulator (ICOS) expression by glomerular leukocytes, which are associated with effector T-cell activation [6] and other T-cell-mediated inflammatory or cytotoxic processes, Refs. [7,8,9,10] have been demonstrated in TG biopsies. We previously demonstrated that TG biopsies without any DSA or C4d displayed alternative molecular signatures suggestive of T cell-mediated immune injury, distinct from that seen in antibody-mediated injury [4,5]. In addition, MVI is not specific for ABMR as it is also observed in other disorders including acute tubular necrosis, glomerulonephritis, and T cell-mediated rejection (TCMR) [11,12]. A recent study by Koenig et al. showed that histologic lesions in half of the patients with MVI are not mediated through antibodies, further demonstrating the role of innate immunity [13]. It is unclear if the driving force in the progression of TG is through antibody-mediated, T cell-mediated mechanisms or innate immunity. T cell recognition of processed alloantigens via the indirect pathway has been shown as a key factor in initiating and maintaining the progression of chronic allograft rejection [14,15,16,17].

Small RNAs (sRNAs) consist of different classes of RNAs such as miRNAs and tRNA fragments. miRNAs are a class of small single stranded noncoding RNAs that are involved in translational repression of specific target mRNAs. They are associated with a myriad of diseases and physiological conditions. tRNA fragments (tRFs) are the new class of small RNAs that are far less studied compared to miRNAs. tRFs are classified into many groups due to variability in size and mapping position on tRNA [18]. tRNA halves are produced by cleavage at the anticodon loop, a process regulated by cellular stress such as hypoxia [19]. tRFs produced from 5′ or 3′ of mature tRNA (tRF-5 or tRF-3), with a similar length to miRNAs, are loaded into Argonaute proteins containing RISC complex and function in a manner similar to that of miRNAs to regulate gene expression [20,21].

In addition to cells and tissues, sRNAs are found in various types of circulating body fluids including serum, plasma and urine [22]. Moreover, miRNAs and tRNA fragments can be found in serum as ribonucleoprotein (RNP) complexes or in extracellular vesicles (EVs) [23]. miRNAs constitute a key regulatory component of immune system development and function. Specific miRNAs impact B-cell and T-cell differentiation, as well as other cellular processes required for innate and adaptive immunity including inflammation, T-cell receptor (TCR) signaling, toll-like receptor (TLR) signaling, cytokine production, T-regulatory (Treg) cell function and antigen presentation. Current evidence suggests that miRNAs are dominant regulators of renal fibrosis [24,25,26]. Moreover, activated T cells secrete certain types of tRFs to mediate the communication between T cells [27].

In this study, circulating sRNAs of kidney transplant patients were explored as a potential non-invasive biomarker for TG and differentially expressed serum miRNAs and tRNA fragments in TG are reported. Biopsies or serum from patients with normal allograft function after transplant were used as a control. miRNAs, tRFs and rRNA fragments were detected in the serum of patients with TG and normal allograft function. The majority of tRFs were generated from 5′ of tRNA, mainly tRNA-Gly and tRNA-Glu. miR-1224-5p, miR-4508, miR-320 and miR-378a were among the downregulated miRNAs in TG serum. Strikingly, downregulation of these miRNAs correlates with the global upregulation of mRNA predicted targets and explains the upregulated canonical pathways such as iCos-iCosL signaling pathway in T helper cells, Th1 and Th2 activation pathway, and dendritic cell maturation, supporting the role of T cell-mediated mechanisms in ongoing allograft injury.

## 2. Results

### 2.1. Clinical Characteristics of the Patients

This study included patients with histopathological diagnosis of TG and available biopsy microarray data and serum samples taken at the time of biopsy. Clinical characteristics of the patients are summarized in Table 1. Fifty six percent are male, 56% are Caucasian, 35% are African American with a median age of 51 (32, 79) years. 68% of the patients received deceased-donor kidney transplantation after a median of 3 (0.7, 13) years being on dialysis. Fifty three percent received anti-thymocyte globulin induction and the median cold ischemia time was 19 h (9, 43). Median HLA mismatch at the time of transplant was 3 (0, 6). Etiology of kidney disease and donor information are summarized in Table 1.

Patients underwent a clinically indicated kidney biopsy at 83 months (3, 276) after kidney transplantation. Twenty four percent of patients had previous history of biopsy-proven acute rejection. At the time of biopsy, 59% had donor-specific anti-HLA antibodies (DSA) with a median class I PRA 10% (0%, 86%) and class II PRA 37% (0%, 99%). However, 24% of the patients did not have any anti-HLA antibodies and had class I and II PRA 0%. Of the 20 patients with DSA, 6 had class I, 9 had class II and 5 had both class I and II DSAs. At the time of biopsy median serum creatinine was 2.0 (1.0, 5.1) mg/dL and 83% had spot urine protein creatinine ratio greater than 0.5 g/g.

The control group consisted of transplant patients who underwent clinically indicated kidney biopsy with completely normal kidney biopsies (all acute and chronic Banff allograft injury scores were zero). Clinical characteristics are summarized in Table 1. The biopsies were done at a median of 4.5 months (1, 120) after transplantation and only 11% had DSAs with 69% having both class I and II PRA 0%. Median serum creatine level was 1.7 mg/dL (1.1, 3.8) and 84% had spot urine protein/creatinine less than 0.5 g/g.

### 2.2. Histopathological Features of TG Biopsies

Thirty two percent of the TG biopsies were C4d positive (C4d score > 0) and 47% had MVI score ≥ 2. Median g score was 1 (0, 2) and ptc score 1 (0, 3). In terms of other acute Banff allograft injury scores, median t score was 0 (0, 1) and i score was 1 (0, 3) and all the biopsies had v scores of 0. In terms of chronic injury scores, median cg score was 1 (1,3), ct score 2 (0, 3), ci score 1 (0, 3), and cv score 1 (0, 3).

### 2.3. Circulating Small RNA Profiles from Serum of Transplant Glomerulopathy and Normal Allograft Transplant Patients

**Figure 1:**
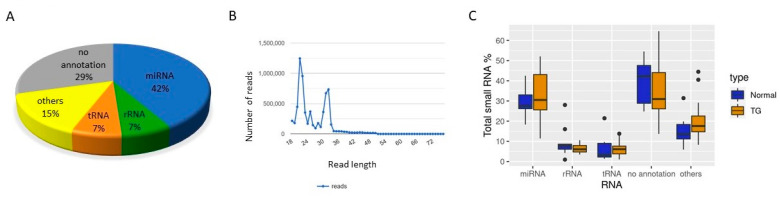
Serum small RNA composition in TG and normal allograft patients. (**A**) Distribution of reads in a representative small RNA library (TG-143). (**B**) Read length distribution of a representative sRNA library. Distinct peaks indicate the protection and cloning of RNA fragments. (**C**) Distribution of different small RNA categories in TG and control patients.

sRNAs from serum of patients with TG (*n* = 25) and with normal allograft function (*n* = 10) were evaluated using next generation sequencing (NGS). Quality control criteria such as percentage of no annotation reads and presence of distinct peaks in the library size distribution were considered, as suggested by exRNA consortium for sequencing data analyses [28]. Serum sRNA population of kidney transplant patients includes different classes of sRNAs and showed two distinct peaks, indicating presence of two major stable sRNA categories as seen in representative library profile (Figure 1A,B). Libraries outside of described criteria were excluded from down-stream analyses. Statistics of libraries, including total number of mapped reads, can be found in Supplementary Table S1.

miRNAs, tRFs and rRNA fragments, as well as fragments from miscellaneous RNAs such as Y RNAs were detected in all studied serum samples. sRNA population consisted of approximately 30% of miRNAs and 5–10% of tRFs and rRNA fragments (Figure 1C). No significant changes in the overall distribution of sRNAs population were observed between serum samples from TG and control groups, which is the case for most diseases. However, downstream analysis identified differentially expressed miRNAs and tRFs in TG compared to control groups.

### 2.4. Abundant 5′ tRNA Fragments in Serum

**Figure 2:**
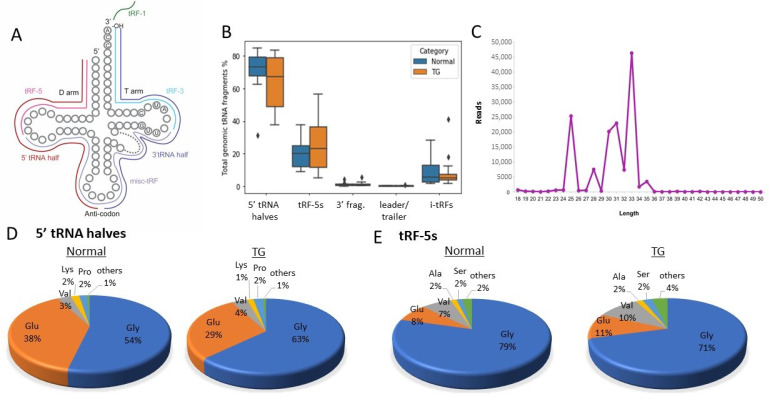
tRNA fragments in TG and normal allograft patients’ serum. (**A**) Schematic showing different classes of tRNA fragments. (**B**) Distribution of tRNA fragment classes in TG and control. (**C**) Length distribution of reads that mapped to 5′ of mature tRNA-Gly-GCC (5′ tRNA fragments) from one of the control libraries. Distinct peaks show presence of tRF-5 and 5′ tRNA halves. (**D**–**E**) Pie charts showing the distribution of 5′ tRNA halves (**D**) and tRF-5 (**E**) based on parental tRNA. Most 5′ tRNA halves and tRF-5s are produced from tRNA-Gly and tRNA-Glu both in TG and control.

Next, the origins of tRFs detected in the serum were determined. tRFs can originate from extreme 5′ ends (tRF-5s or 5′ tRNA halves if cut in the anticodon loop), extreme 3′ ends with or without CCA (tRF-3s or 3′ tRNA halves if cut in the anticodon loop), 3′ trailer sequence (tRF-1) and anywhere on tRNA but not mapping to extreme 5′ or 3′ ends (internal fragments) (Figure 2A). In the patient serum, the majority of tRNA fragments (50–80% of tRNA fragments) were 5′ tRNA halves (Figure 2B). The second most abundant class was tRF-5s (10–30% of total tRNA fragments). The length of these 5′ tRFs in individual libraries can be visualized as distinct peaks, indicating that these fragments are processed (rather than randomly degraded products) (Figure 2C), supporting lack of false discoveries. Although there were changes in the median value of 5′ tRNA halves percentages between TG and control groups, the difference was not statistically significant. Internal fragments range from 3–20%. Small percentages of tRFs also mapped to 3′ of tRNA and leader or trailer sequences of the tRNA.

We then explored the specific tRNA origins of the most abundant fragment class: the 5′ tRNA halves. As shown in Figure 2D, more than 90% of 5′ tRNA halves in the serum of transplant patients originated from tRNA-Gly (50–60%) or tRNA-Glu (30–40%). As reported by Godoy et al. [22] the distribution of tRFs to certain tRNAs is similar in healthy individuals’ serum as well. Likewise, tRF-5s mainly originate from tRNA-Gly (~70%) followed by tRNA-Glu (~10%) and tRNA-Val (8–10%) (Figure 2E).

### 2.5. Differentially Expressed tRNA Fragments in TG Patients

**Figure 3:**
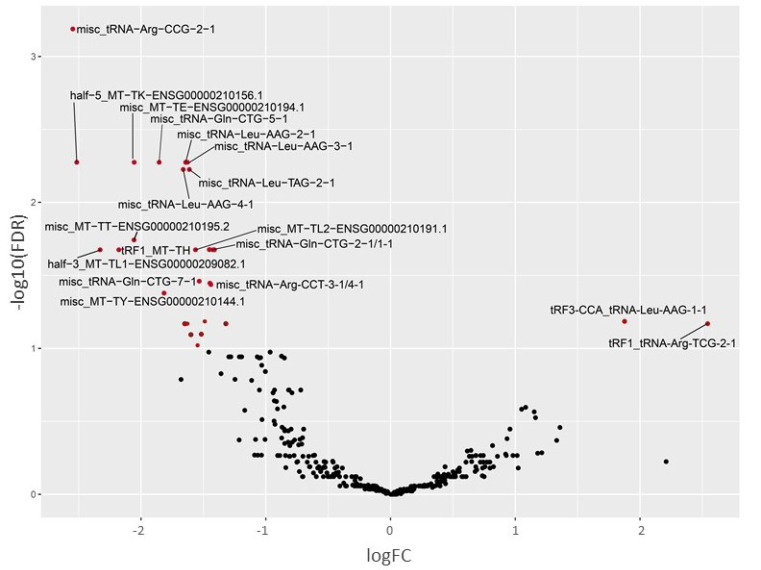
Volcano plot showing the differentially expressed tRNA fragments in TG compared to normal allograft. Significant changes with LFC2 > 1 with FDR < 0.1 are labeled red.

We identified 30 differentially expressed tRFs in patients with TG compared to normal allograft function (log2 fold change (LFC2) > 1, FDR < 0.10) (Figure 3 and Supplementary Table S2). Most of them are classified as internal tRNA fragments coming from the internal parts of mature tRNA, not mapping to extreme 5′ or 3′ of mature tRNA (Supplementary Table S2). Downregulated 5′ tRNA halves (half-5_tRNA-Leu-AAG-3-1, half-5_tRNA-Leu-TAG-1-1) and 3′ tRNA halves (half-3_tRNA-Glu-TTC-3-1) as well as tRFs mapping to mitochondrial tRNA genes were also detected. Both tRNA-halves and internal-tRFs have been reported to be involved in translational repression [29].

### 2.6. Differentially Expressed miRNAs in TG Patients

**Figure 4:**
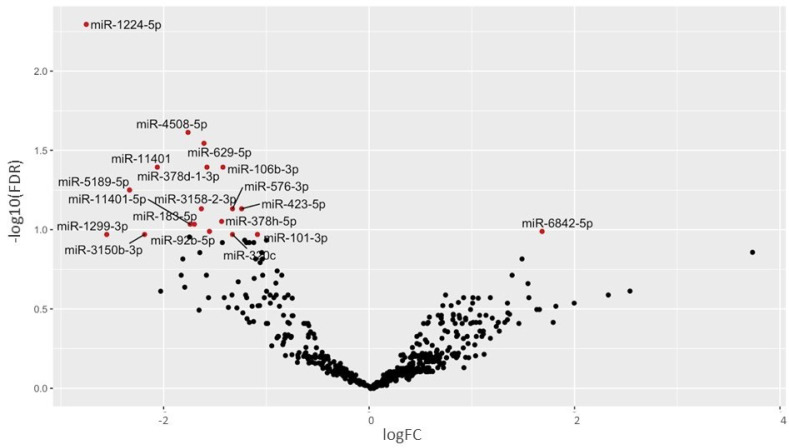
Volcano plot showing the differentially expressed miRNAs in TG compared to normal allograft function. Significant changes with logFC > 1 with FDR ≤ 0.1 are labeled red.

In the studied serum samples, miRNAs were the most abundant category (~30% of sRNA pool) (Figure 2C). Nineteen miRNAs were differentially expressed between TG and control serum samples (FDR ≤ 0.1) (Figure 4, Supplementary Table S3). Interestingly, 18 miRNAs were downregulated in the serum of TG. Some of the significantly differentially expressed miRNAs were found to be related to kidney development or pathology of kidney disease indicating the findings are related to condition [30,31,32,33].

As it was observed that some samples behaved as outliers in PCA (Supplementary Figure S1), differential expression analysis after removal of outliers (N_97, N_165, N_246, TG_54, TG_149, and TG_422) was performed. The new analysis identified a greater number of differentially expressed miRNAs including the 19 differentially expressed miRNAs that were identified using the complete sample set (Supplementary Figure S2 and Supplementary Table S4).

### 2.7. Down Regulation of Circulating miRNAs Results in Upregulation of Targets Globally

**Figure 5:**
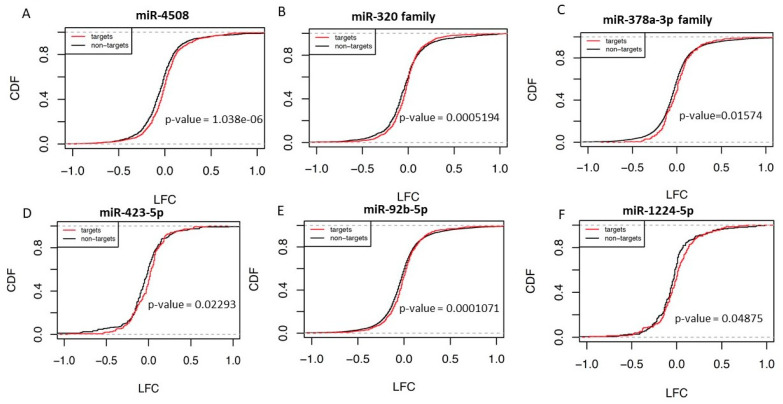
Cumulative distribution plots of mRNAs based on miRNA target status. mRNA targets of downregulated serum miRNAs (miR-4508 (**A**), miR-320 (**B**), miR-378a-3p (**C**), miR432-5p (**D**), miR-92b-5p (**E**) and miR-1224-5p (**F**)) are upregulated in the kidney tissue. mRNA targets of miRNA are predicted using TargetScan based on seed sequence complementarity. Non-targets represent mRNA which do not have complimentary seed match with the miRNA.

Publicly available kidney graft gene expression from this set of patients (GSE93659) was integrated with circulating miRNA data to demonstrate potential function of serum miRNAs and further evaluate cellular interactions and inter-cellular communication in TG. 232 genes were upregulated while 38 were downregulated (LFC2 0.6, FDR 0.05) in the kidney tissues with TG (Supplementary Table S5). We checked cumulative distribution function (CDF) of mRNA targets of differentially expressed miRNAs using gene expression data. As shown in Figure 5, predicted targets of downregulated miR-1224-5p and miR-4508 were upregulated (shift of the target mRNAs compared to non-target mRNAs to right indicates overexpression of mRNAs, which are targets of given miRNAs). miRNA target recognition was evaluated based on the published criteria [34,35]. In addition to these two miRNAs, we also explored miR-320, miR-378 (miR-378d and miR-378h), miR-423-5p and miR-92b which are downregulated in TG and were shown to have a role in kidney function [32,36]. Strikingly, CDF plots for the targets of miR-320, miR-378, miR-423-5p and miR-92b showed a right shift indicating upregulation in the targets of miR-320, miR-378, miR-423-5p and miR-92b, respectively (Figure 5B–E).

### 2.8. Circulating miRNAs Regulate Top Pathways Identified in Gene Expression Analysis

**Figure 6:**
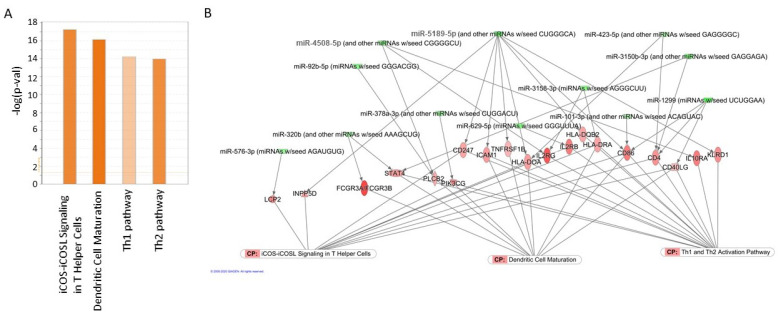
Pathway integration of miRNAs and mRNA changes using IPA. (**A**) Canonical pathways enriched in TG based on gene expression changes using IPA. (**B**) Changes in miRNAs can explain the changes in canonical pathways. Green: Repression Red: Activation.

miRNA and mRNA datasets (mRNA available for same set of samples (GSE93659) were also integrated and subjected to network analyses by Ingenuity Pathway Analysis (IPA) to identify plausible associations and potential regulatory networks relating to TG. The top upregulated canonical pathways in TG biopsies included iCos-iCosL signaling pathway in T helper cells (z-score: 3.5), dendritic cell maturation (z-score: 4.025), and Th1 (z-score: 1.667) and Th2 pathways (z-score: 3.317) (Figure 6A). Furthermore, differentially expressed miRNAs and mRNAs were integrated using IPA tools. These analyses showed that downregulated miRNAs in the serum of TG patients are potential upstream regulators of overexpressed genes in the kidney tissues. These up-stream regulators explain the changes leading to activated canonical pathways such as iCOS-iCOSL signaling in T helper cells, dendritic cell maturation and Th1 and Th2 pathways (Figure 6B). It is important to note that our integration was done using experimentally identified differentially expressed miRNAs and mRNAs from TG patients. Furthermore, using a small validation set, we were able to confirm trends of the down-regulation of serum miR-101-3p (*p* = 0.00768) and miR-320b (*p* = 0.0612) (Supplementary Figure S3). The expression of these miRNAs and others needs to be further investigated using a larger cohort.

## 3. Discussion

In this study, we characterized circulating sRNA profiles in the serum of transplant patients with TG and normal allograft function using NGS. To our knowledge, this is the first study to evaluate circulating sRNA profiles in kidney transplant patients with TG. Moreover, this study includes an integrative approach by combining circulating sRNA and tissue graft gene expression profiles to elucidate potential up-stream regulators of critical molecular pathways associated with TG. Although limited by a small sample size, our study takes advantage of the combined analyses from unique graft biopsies with TG and serum samples. Altogether, our results demonstrate that sRNA profiles reflect the status of patients and are potential non-invasive biomarkers for TG. Moreover, changes in serum miRNAs and tissue gene expression support the role of T-cell mediated mechanisms in kidney allograft injury and pathogenesis of TG.

The extracellular/circulating small RNA sequencing profiles detected several different classes of small RNAs, with miRNAs being the most abundant class followed by rRNA and tRNA fragments, respectively. This is consistent with previous studies [22,37]. However, recent studies also have shown that detection of tRFs are highly abundant in extracellular environment [38]. tRNAs contain multiple modifications which may interfere with library preparations resulting in the lower representation of tRFs [39]. The majority of tRFs detected in the serum of transplant patients originate from 5′ of mature tRNA, with ~50–70% originating from tRNA-Glycine. Interestingly, it has been reported that fragments arising from tRNA-Gly and tRNA-Glu are more stable which results in higher abundances in small RNA sequencing libraries [40,41]. There were 30 differentially expressed tRFs in the serum of TG compared to control group. Most of the differentially expressed tRFs were 5′ tRNA halves and internal tRFs, mainly upregulated under stress conditions [42]. tRFs are potential mediators of immune responses as they have been implicated in immune associated responses such as viral infections [43]. Additionally, 5′ tRNA halves activate Toll-like receptor 7 (TLR7) upon *Mycobacterium tuberculosis* infection [44]. Zhang et al. demonstrated that tRNA half generated from tRNA-Glu downregulates the expression of CD1a in dendritic cells [45]. Interestingly, Chiou et al. showed that activated T cells release extracellular vesicles which are enriched in tRF-5s but depleted in tRF-3s. Selective release of tRFs in extracellular vesicles results in inhibition of T cell activation through a feedback loop [27]. Moreover, tRFs regulate T cell receptor signaling pathway, Th1 and Th2 cell differentiation and primary immunodeficiency in systemic lupus erythematosus [46]. More recently, Winek et al. showed that the balance between miRNAs and tRFs are important in poststroke immune blockade [47]. Herein, a signature of circulating tRNA fragments in TG is presented for the first time. The role of tRFs to tune translation and control protein expression upon cellular stress during kidney injury is in its infancy [48,49]. Our results represent the foundation for further studies to explore tRFs as a non-invasive TG biomarker for clinical diagnostic applications.

Nuclease resistant extracellular sRNAs have been found in all known biological fluids, predominantly as in RNPs or EVs. The biological function of extracellular miRNAs remains uncertain; however, strong evidence suggests that extracellular miRNAs carry cell–cell signaling function throughout various physiological and pathological processes [50,51]. In this study, we identified 18 downregulated miRNAs in TG compared to normal allograft function group, including miR-320, miR-378a-3p, miR-423-5p, miR-92b-5p and miR-101. Previous studies showed that these miRNAs have pertinent functions in the kidney. For example, miR-320 plays an important role in IgA nephropathy by promoting B cell proliferation through suppression of PTEN expression [52]. Additionally, miR-378-3p was implicated in glomerular disease and kidney tubular fibrosis by regulating nephronectin and MAPK signaling, respectively [32]. miR-423-5p modulates the activation of NF-kB by targeting TNIP2 and contributes to the pathogenesis of lupus nephritis [33]. Moreover, low miR-101-3p serum levels are associated with acute kidney injury [53].

Notably, gene expression (GSE93659) from the same set of patients’ biopsies collected at the same time as serum was analyzed with an unbiased genome-wide approach and showed the importance of immunological response to graft as seen canonical pathways in IPA. These analyses demonstrated a role for adaptive immune response in the TG group. We previously reported glomerular infiltration by CXCR3+ ICOS+ activated T cells in chronic allograft nephropathy with TG [6]. In a later publication, we suggested that while DSA+/C4d− TGP biopsy specimens may be classified as CAMR, DSA−/C4d− TGP specimens showed increased cytotoxic T cell-associated transcripts, suggesting T cell activation as a mechanism of injury [4]. Koenig et al. recently reported a type of chronic rejection, whose pathophysiology is independent of the recipient’s adaptive immune system and the role of innate immunity [13]. TG leads to an end-organ damage that most often develops through ABMR in concert with cellular immune responses, innate immunity, endothelial damage from infections (viruses) or drugs. Although 59% of patients had DSA, 32% had C4d and 47% MVI, suggesting that the main driving force for allograft injury was through T cell-mediated mechanisms.

Critically, our integrative approach showed that downregulated serum miRNAs result in the upregulation of the target mRNAs in the kidney tissue. Recent reports support our approach and results by showing a correlation between circulating miRNAs and tissue gene expression. Specifically, evidence for functional cell-to-cell miRNA transfer was found during investigation of the immune synapse formation. Mittelbrunn et al. showed that exosomes of T, B, and dendritic immune cells contained different miRNA repertoires. Furthermore, miRNAs were transported from T cells to antigen presenting cells unidirectionally through an antigen-driven transport mechanism [54]. Using in vivo and in vitro models, Spinosa et al. reported the critical role of MSC-derived EVs in attenuation of aortic inflammation and macrophage activation via miR-147 during abdominal aortic aneurysm formation [55]. As the study of circulating miRNA mediated cell-cell signaling in mammals advances, the path to clinical translation and application becomes increasingly more apparent.

In summary, this is the first study to report the circulating sRNA profiles in transplant patients with TG using NGS. Patients with TG exhibited a novel circulating tRNA fragment signature in their serum. Integration of serum miRNA profiles with tissue gene expression demonstrated the role of T-cell mediated mechanisms where circulating miRNAs are potential upstream regulators in ongoing allograft injury. To validate our results, a multicenter study with large numbers of biopsy and serum specimens needs to be conducted. However, we present the foundation for further exploration and validation of potential non-invasive biomarkers that align with disease graft biology by showing the feasibility of sRNA evaluation from serum samples using high throughput approaches.

## 4. Materials and Methods

Patients who were enrolled in the IRB-approved “Immune Monitoring Study” with clinically indicated biopsy samples and blood samples were used for analysis. Clinical data was collected on all patients by chart review. This study was approved by the Montefiore/Einstein Institutional Review Board (09-06-174). The clinical and research activities being reported are consistent with the Principles of the Declaration of Istanbul as outlined in the ‘Declaration of Istanbul on Organ Trafficking and Transplant Tourism’.

### 4.1. Histopathology

Biopsies were examined by light microscopy using hematoxylin and eosin, periodic acid-Schiff (PAS), Masson Trichrome and C4d immunoperoxidase stains. Immunoperoxidase staining for C4d was performed on paraffin embedded sections using a polyclonal rabbit anti-human antibody (Cell Marque) at a dilution of 1:100 with the Dako Envision system. Evaluation of the biopsies was based on the Banff acute and chronic indices including glomerulitis (g), interstitial inflammation (i), tubulitis (t), intimal arteritis (v), peritubular capillaritis (ptc), transplant glomerulopathy (cg), mesangial matrix increase (mm), interstitial fibrosis (ci), tubular atrophy (ct), and vascular fibrous intimal thickening (cv) [56], as well as microvascular inflammation score (mvi) = (g) + (ptc) [57]. Biopsies were diagnosed as TG by electron microscopy if they showed electron-lucent widening of the subendothelial zone of the glomerular basement membrane or subendothelial accumulation of flocculent material, with or without a new subendothelial basement membrane layer.

### 4.2. RNA Purification from Serum and sRNA Library Preparation

Small RNAs were purified from 200 µL serum using Qiagen miRNeasy Serum/Plasma Advanced kit (Cat No. 217204) and eluted with 15 µL RNase free water. sRNA library was made with 5 µL of RNA using NEBNext small RNA library Prep Set (Cat No. E7330L) according to the manual. 18 cycles of PCR amplification were performed. sRNA libraries were purified by polyacrylamide gel and subjected to Agilent bioanalyzer. Equal amounts of libraries were mixed and sequenced in NextSeq500 in Oklahoma Medical Research Foundation.

### 4.3. sRNA Sequencing Data and Differential Expression Analysis

sRNA sequencing was done for 10 normal and 25 TG patients. The libraries with more than 50% of un-annotated reads (possible DNA contamination/degradation) were considered as bad quality libraries and were removed for further analysis. Specifically, the length distribution for mapped reads with no distinct peaks indicating possible RNA degradation are excluded from analysis. 9 control and 21 TG libraries were then considered for differential expression analysis. The data can be found at GSE156874. The details of mapping and differential expression analysis are described below.

For small RNA-seq, unitas (version unitas_1.7.0) was used to cut adaptor AGATCGGAAGAG and reads smaller than 18 were filtered out. To quantify small RNAs, unitas v1.7.0 [58] (with SeqMap v1.0.12 [59]) was used to map the reads to human sequence of miRBase Release 21 [60], genomic tRNA database [61], Ensembl Release 88 [62] and SILVA rRNA database Release 128 [63]. Unitas setting (–trim_minlength 18 –species homo_sapiens –trim AGATCGGAAGAG) was used to find different small RNA species. This setting is equivalent to –tail 2 –intmod 1 –mismatch 1 –insdel 0 which will allow 2 non-templated 3′ nucleotides and 1 internal mismatch for miRNA mapping and 1 mismatch, 0 insertion/deletion for tRNA fragments mapping. For differential analysis, edgeR was used on count matrix of tRFs and miRNAs. filterByExpr function of edgeR is used to keep rows that have worthwhile counts in a minimum number of samples. The calcNormFactors function of edgeR is used to calculate effective library size and to normalize for RNA composition by finding trimmed mean of M-values (TMM) between each pair of samples. Tagwise and common dispersions were estimated using the estimateDisp function. miRNAs and tRFs, which are expressed more than 1 count per million reads in at least 10 samples, were considered for the finding of differentially expressed miRNAs and tRFs.

### 4.4. Differential Gene Expression Analysis Using Microarray Data

Gene expression data from kidney biopsies is publicly available in GEO (GSE GSE93659). The Affymetrix Detection Call algorithm was used to determine whether probe sets were present, marginally present, or absent in each sample. The percentage of present calls and the 3′:5′ ratio for GAPDH for each sample was examined [64]. To obtain probe set expression summaries, we used the robust multiarray average method [65]. Prior to statistical analysis the gene expression data matrix was filtered to exclude probe sets called absent in all samples and control probe sets. 

### 4.5. miRNA Target Prediction and Cumulative Distribution Plots

Targets of top differentially expressed miRNA (miR-1224-5p, miR-4508) and miRNA obtained by Ingenuity Pathway Analysis (IPA) analysis (miR1285-3p, miR-378a-3p, miR-92b-5p) were predicted using TargetScanHuman Release 7.2 [66]. Cumulative distribution function was used to compare the fold-change between target and non-target genes of each miRNA. Kolmogrov–Smirnov test in R was used to evaluate if the log2 fold change of target genes was significantly increased in TG patients compared to that of non-targets genes.

### 4.6. Integration of miRNA and Gene Expression Analysis

Ingenuity Pathway Analysis (IPA) (Qiagen) was used to integrate the miRNA and gene expression data. Specifically, mRNA targets of significant miRNAs were determined using IPA’s miRNA Target Filter, which identifies experimentally validated miRNA-mRNA interactions from TarBase, miRecords, and the peer-reviewed biomedical literature, as well as predicted miRNA-mRNA interactions from TargetScan. A conservative filter was applied using only experimentally validated and highly conserved predicted mRNA targets for each miRNA, as identified by TargetScan within the IPA software. Highly conserved pairings are predicted by TargetScan to repress expression of mRNA target to <40% of “normal” levels. These mRNA targets were carried through to Core Pathway Analyses, which identified common pathways containing the mRNAs in our data set. As noted above, *P* values were assigned to pathways via a Fischer exact test to reduce the risk of false positive findings from the original ANOVA, as pathway components represent interrelated rather than independent elements. Canonical pathways, novel networks, and common upstream regulators were then queried for overlap with targets from our differentially expressed miRNA gene target list.

## Figures and Tables

**Table 1:** Demographic information of the study groups.

Transplant Glomerulopathy (*n* = 34)		Control Group (*n* = 19)
Median age	51 (32, 79)	56 (27, 71)
Sex, male	56%	79%
Race, Caucasian	56%	39%
Etiology of kidney disease		
Diabetes Mellitus	21%	37%
Hypertension	29%	32%
Glomerular disease	32%	16%
Median time on dialysis, yr	3 (0.7, 13)	3 (1, 8)
Type of transplant, deceased	68%	89%
Median donor age	37 (5, 63)	36 (6, 72)
Donor sex, male	47%	63%
Donor race, Caucasian	44%	53%
Median Class I PRA	10% (0%, 86%)	0% (0%, 97%)
Median Class II PRA	37% (0%, 99%)	0% (0%, 100%)
Both Class I and II PRA 0%	24%	69%
Median HLA mismatch	3 (0, 6)	3 (0, 6)
Donor-specific antibody	59%	11%
Induction, anti-thymocyte	53%	63%
Median cold ischemia time	19 (9, 43) h	21 (9, 45) h
Median time to biopsy		
after transplantation	87 (3, 276) mos	4.5 (1, 120) mos
Previous acute rejection	24%	0%
Median serum creatinine	2.0 (1.0, 5.1) mg/dL	1.7 (1.1, 3.8) mg/dL
Spot urine protein/creatinine		
more than 0.5 g/g	83%	16%

## Data Availability

Gene expression by microarrays are publicly available in GEO, GSE93659. sRNA profiles from serum of TG and normal allograft function patients are available at GEO, GSE156874.

## Supplementary Materials

The following are available online at https://www.mdpi.com/article/10.3390/ijms22126218/s1. Figure S1: PCA plot including all studied samples, Figure S2. Differentially expressed miRNAs after excluding outliers, Figure S3: RT-qPCR validation of miRNAs using serum samples from patients with TG and Normal Allograft Function, Table S1: Mapping statistics of small RNA sequencing, Table S2: Differentially expressed tRNA fragments in TG compared to normal allograft function, Table S3: Differentially expressed miRNAs in TG compared to normal allograft function, Table S4: Differentially expressed miRNAs in TG compared to normal allograft function. Table S5: Differentially expressed genes in TG compared to normal allograft function.
