# Supplementary material for: Integrative Analyses of Circulating Small RNAs and Kidney Graft Transcriptome in Transplant Glomerulopathy
Source: Int J Mol Sci. 2021 Jun 9;22(12):6218. doi: 10.3390/ijms22126218 (PMC8226568; doi:10.3390/ijms22126218)
Supplement: Supplementary file 1 [file ijms-22-06218-s001.zip › ijms-1202120-supplementary.pdf]

Integrative analyses of circulating small RNAs and kidney graft transcriptome in transplant glomerulopathy

Canan Kuscu[1, †, ‡], Manjari Kiran[2, †], Akram Mohammed[3], Cem Kuscu[1], Sarthak Satpathy[2], Aaron Wolen[1], Elissa Bardhi[4], Amandeep Bajwa[1], James D. Eason[1], Daniel Maluf [1,4], Valeria Mas[1,4,&], Enver Akalin[5,&]

[1] Transplant Research Institute, James D. Eason Transplant Institute, Department of Surgery, School of Medicine, University of Tennessee Health Science Center, Memphis, TN

[2] Department of Systems and Computational Biology, School of Life Sciences, University of Hyderabad, Hyderabad, India

[3] Center for Biomedical Informatics, University of Tennessee Health Science Center, Memphis, TN

[4] Department of Surgery, School of Medicine, University of Maryland, Baltimore, MD

[5] Montefiore Medical Center, Abdominal Transplant Program, Albert Einstein College of Medicine, Bronx, NY

† Equal contribution
‡ Corresponding author
& Shared senior authorship

Correspondence:
Canan Kuscu, PhD
University of Tennessee Health Science Center
Department of Surgery
Transplant Research Institute, James D. Eason Transplant Institute
71 S Manassas St. TSRB Rm 421
Memphis, TN 38103
901-448-3162
ckuscu@uthsc.edu

Supplementary Figures:

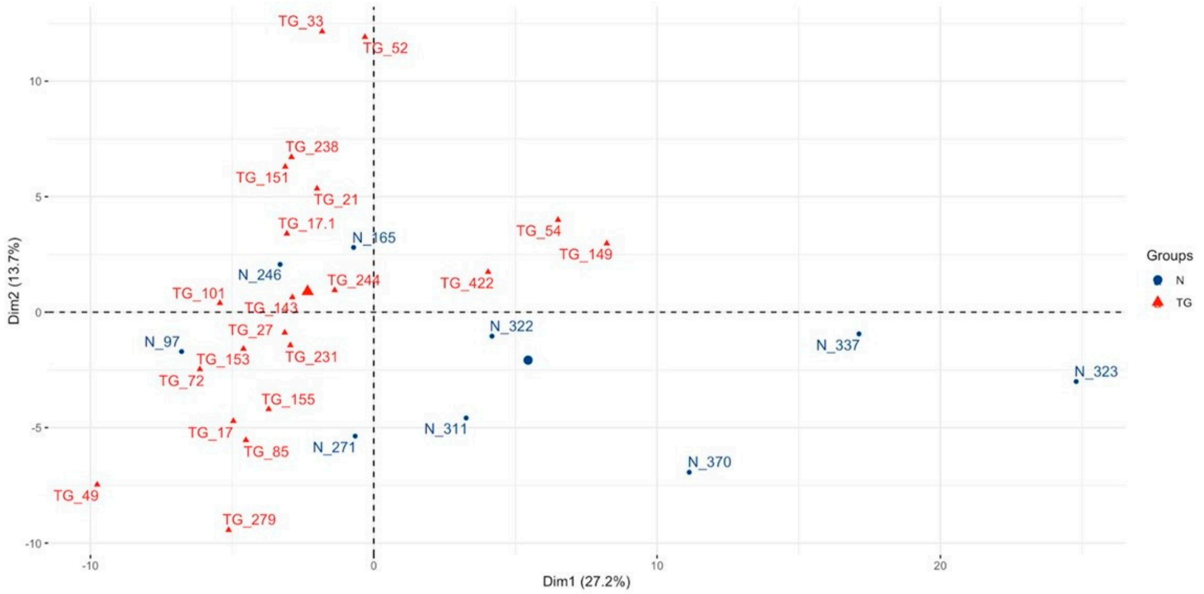

Supplementary Figure S1. PCA plot using miRNAs showing the variability in the patient samples.

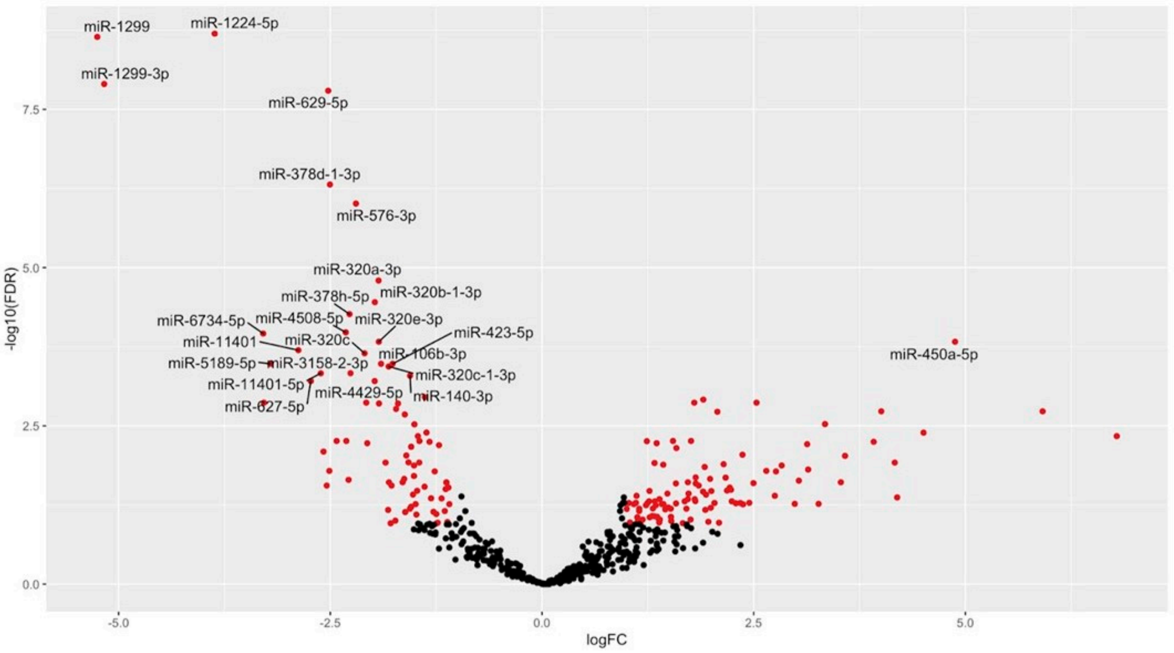

Supplementary Figure S2. Differentially expressed miRNAs after excluding outliers. Volcano plot showing the differentially expressed miRNAs after removing outliers (N_97, N_165, N_246, TG_54, TG_149, and TG_422. Red: LFC> 1 and FDR <0.1, Labeled: LFC> 1 and FDR<0.001.

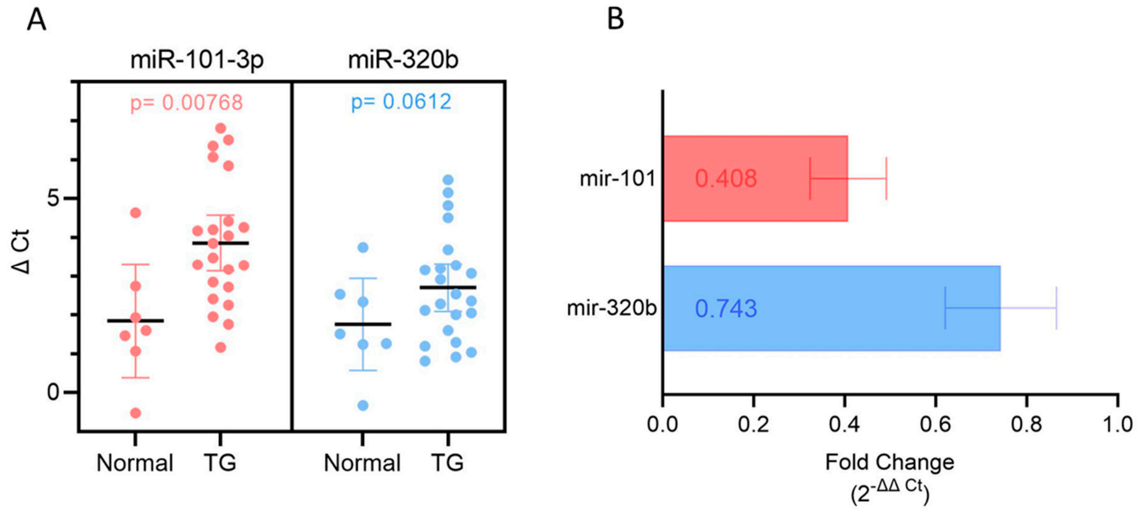

Supplementary Figure S3. A. Independent RT-qPCR validation of 2 candidate miRNAs using serum from TG (n=22) and Normal (n=7) patients. Plotted with mean values and 95% confidence intervals. Gene expression normalized using U6 snRNA housekeeping gene to calculate ΔCt. **B.** Average Log2 Fold Change values for RT-qPCR expression using the $2^{-\Delta\Delta Ct}$ method, depicted with the standard error of the mean. ΔΔCt values were calculated using the mean values of the Normal groups for each miRNA.

Supplementary tables

Supplementary Table S1: Mapping statistics of small RNA sequencing

	Total_Read	miRNA	rRNA	tRNA	others	no annotation
**N_97**	6359168	1164874	388000	613554	1166846	3025894
**N_165**	4462155	1169118	189296	400353	1401475	1301913
**N_246**	10564242	4488619	820561	619827	1836873	2798362
**N_271**	2998685	988085	27938	642980	595835	743847
**N_311**	5251652	1733740	377985	116449	711223	2312255
**N_322**	5570625	1958078	472181	166979	624087	2349300
**N_323**	3821225	847742	612484	51682	225664	2083653
**N_337**	6425275	1761164	1800187	209782	800095	1854047
**N_370**	6012907	1652282	515711	113939	608727	3122248
**TG_17**	3959251	517898	403643	40133	440568	2557009
**TG_21**	3916361	446787	412919	67854	1010761	1978040
**TG_27**	4213715	968524	333391	442919	975660	1493221
**TG_33**	5279674	1459330	265933	401949	2139398	1013064
**TG_49**	6181404	1785526	493865	415121	731543	2755349
**TG_52**	3192515	967747	219431	160812	484997	1359528
**TG_54**	5287933	1612803	320637	64487	1021408	2268598
**TG_72**	6439485	2201327	423912	808755	1012692	1992799
**TG_85**	8725665	4546269	377078	609708	1967388	1225222
**TG_101**	3925950	1690863	183832	176794	779551	1094910
**TG_143**	5343160	2224602	383931	378335	784748	1571544
**TG_149**	3924484	1004479	326988	148653	578381	1865983
**TG_151**	7572242	1912784	260325	994276	3367282	1037575
**TG_153**	10101104	4782425	425995	479551	1776354	2636779
**TG_155**	10919806	5459006	425568	866692	1766393	2402147
**TG_176**	4159568	1898012	226673	263612	618099	1153172
**TG_231**	3967621	934779	296689	96119	716451	1923583
**TG_238**	5330497	1680404	251205	325229	1552943	1520716
**TG_244**	6881050	2762099	383764	948665	1400615	1385907
**TG_279**	4733497	2245947	286568	51774	391999	1757209
**TG_422**	5540439	1611082	536885	237179	714441	2440852

Supplementary Table S2: Differentially expressed tRNA fragments in TG compared to normal allograft function

	logFC	logCPM	PValue	FDR
**misc_tRNA-Arg-CCG-2-1**	-2.5473	5.639941	6.91E-07	0.000648
**misc_tRNA-Gln-CTG-5-1**	-1.85492	7.770935	3.48E-05	0.005315
**misc_MT-TE-ENSG00000210194.1**	-2.05516	7.259663	3.53E-05	0.005315
**misc_tRNA-Leu-AAG-2-1**	-1.64212	5.667175	4.02E-05	0.005315
**misc_tRNA-Leu-AAG-3-1**	-1.62807	5.622397	4.75E-05	0.005315
**half-5_MT-TK-ENSG00000210156.1**	-2.51566	4.852916	5.1E-05	0.005315
**misc_tRNA-Leu-TAG-2-1**	-1.6129	5.619937	6.61E-05	0.005951
**misc_tRNA-Leu-AAG-4-1**	-1.66189	5.457052	6.98E-05	0.005951
**misc_MT-TT-ENSG00000210195.2**	-2.05646	2.8503	0.000232	0.018101
**misc_tRNA-iMet-CAT-1-1**	-1.45262	9.12127	0.000464	0.021081
**misc_tRNA-iMet-CAT-2-1**	-1.45067	9.11475	0.000472	0.021081
**misc_MT-TL2-ENSG00000210191.1**	-1.56343	4.866853	0.000595	0.021144
**tRF1_MT-TH**	-2.17833	3.10422	0.000616	0.021144
**half-3_MT-TL1-ENSG00000209082.1**	-2.32825	2.941172	0.000628	0.021144
**misc_tRNA-Gln-CTG-2-1**	-1.42289	8.090903	0.000674	0.021144
**misc_tRNA-Gln-CTG-1-1**	-1.41207	8.103048	0.000676	0.021144
**misc_tRNA-Gln-CTG-7-1**	-1.53353	6.556387	0.001144	0.034629
**misc_tRNA-Arg-CCT-3-1**	-1.44779	9.389731	0.001219	0.035725
**misc_tRNA-Arg-CCT-4-1**	-1.44025	9.393568	0.001284	0.036503
**misc_MT-TY-ENSG00000210144.1**	-1.81554	2.818386	0.001515	0.041796
**misc_tRNA-Gln-TTG-2-1**	-1.48912	6.422396	0.002627	0.06528
**tRF3-CCA_tRNA-Gln-TTG-1-1**	1.876155	3.05902	0.002714	0.06528
**half-5_tRNA-Leu-AAG-1-1**	-1.64934	3.470969	0.003471	0.067723
**tRF1_tRNA-Arg-TCG-2-1**	2.542131	1.94371	0.003613	0.067723
**half-5_tRNA-Leu-TAG-1-1**	-1.62864	3.483088	0.003617	0.067723
**misc_tRNA-Gln-TTG-3-1**	-1.31983	6.585682	0.00382	0.067723
**half-3_tRNA-Glu-TTC-1-1**	-1.51694	3.234167	0.004687	0.079929
**misc_tRNA-Leu-AAG-1-1**	-1.60056	3.539803	0.004979	0.080521
**misc_tRNA-Leu-TAG-1-1**	-1.54694	3.56666	0.005993	0.09528
**tRF1_tRNA-Gly-CCC-3-1**	-1.45698	1.989538	0.006847	0.106076
**half-5_tRNA-iMet-CAT-1-1**	-0.96501	6.424261	0.007803	0.106076

Supplementary Table S3: Differentially expressed miRNAs in TG compared to normal allograft function

	logFC	logCPM	PValue	FDR
**hsa-miR-1224-5p**	-2.75	5.32	9.30E-06	0.00506149
**hsa-miR-1299-3p**	-2.55	7.32	3.41E-03	0.107253479
**hsa-miR-5189-5p**	-2.33	3.85	7.23E-04	0.056192526
**hsa-miR-3150b-3p**	-2.19	4.15	3.49E-03	0.107253479
**hsa-miR-11401**	-2.06	4.02	4.01E-04	0.040398896
**hsa-miR-4508-5p**	-1.76	7.87	8.96E-05	0.024358817
**hsa-miR-11401-5p**	-1.74	5.13	2.12E-03	0.092321095
**has-miR-183-5p**	-1.70	9.60	2.21E-03	0.092321095
**hsa-miR-3158-2-3p**	-1.63	8.31	1.29E-03	0.073704143
**hsa-miR-629-5p**	-1.61	10.96	1.58E-04	0.02856701
**hsa-miR-378d-1-3p**	-1.58	7.70	4.46E-04	0.040398896
**hsa-miR-92b-5p**	-1.55	4.86	2.83E-03	0.102605115
**hsa-miR-378h-5p**	-1.44	6.79	1.79E-03	0.088661907
**hsa-miR-106b-3p**	-1.42	10.51	3.51E-04	0.040398896
**hsa-miR-320c**	-1.33	7.88	3.75E-03	0.107253479
**hsa-miR-576-3p**	-1.33	7.96	1.29E-03	0.073704143
**hsa-miR-423-5p**	-1.24	15.01	1.35E-03	0.073704143
**hsa-mir-101-3p**	-1.09	10.36	3.63E-03	0.107253479
**hsa-miR-6842-5p**	1.68	2.43	2.76E-03	0.102605115

Supplementary Table S4: 19 differentially expressed miRNAs are still differentially expressed after removal of outliers.

	logFC	logCPM	PValue	FDR
**hsa-miR-1224-5p**	-3.86507	5.402486	3.67E-12	2.01E-09
**hsa-miR-1299-3p**	-5.17016	7.116153	6.88E-11	1.26E-08
**hsa-miR-629-5p**	-2.52503	10.90149	1.17E-10	1.60E-08
**hsa-miR-378d-1-3p**	-2.50394	7.619851	4.44E-09	4.86E-07
**hsa-miR-576-3p**	-2.19706	7.845813	1.06E-08	9.72E-07
**hsa-miR-378h-5p**	-2.27309	6.733911	8.92E-07	5.43E-05
**hsa-miR-4508-5p**	-2.31742	7.839453	1.92E-06	0.000105
**hsa-miR-11401**	-2.87693	4.030772	5.15E-06	0.000202
**hsa-miR-320c**	-2.09546	7.85314	6.18E-06	0.000226
**hsa-miR-423-5p**	-1.76436	15.06639	1.01E-05	0.000331
**hsa-miR-5189-5p**	-3.20812	3.852696	1.07E-05	0.000331
**hsa-miR-106b-3p**	-1.90058	10.57198	1.09E-05	0.000331
**hsa-miR-3158-2-3p**	-2.26138	8.398404	1.70E-05	0.000466
**hsa-miR-11401-5p**	-2.61205	5.080074	1.79E-05	0.000467
**hsa-miR-92b-5p**	-2.07493	4.959851	7.07E-05	0.00136
**hsa-miR-3150b-3p**	-3.2842	4.119676	7.32E-05	0.00136
**hsa-miR-101-3p**	-1.46505	10.43687	0.000356	0.004597
**hsa-miR-183-5p**	-2.06342	9.70315	0.000566	0.005942
**hsa-miR-6842-5p**	2.185094	2.334294	0.006137	0.033299

Supplementary Table S5: Differentially expressed genes in TG compared to normal allograft function

ensemblg	symbol	logFC	P.Value	adj.P.Val
ENSG00000138798	**EGF**	-2.13984	1.99E-04	0.021216
ENSG00000198398	**TMEM207**	-1.59217	2.81E-04	0.023072
ENSG00000139988	**RDH12**	-1.5872	1.05E-04	0.016302
ENSG00000113905	**HRG**	-1.44469	8.91E-05	0.015287
ENSG00000169550	**MUC15**	-1.41631	8.34E-04	0.036759
ENSG00000088926	**F11**	-1.26385	4.07E-04	0.028118
ENSG00000117115	**PADI2**	-1.19289	1.09E-04	0.01678
ENSG00000137872	**SEMA6D**	-1.19284	1.46E-04	0.019237
ENSG00000171004	**HS6ST2**	-1.06506	5.62E-05	0.012539
ENSG00000165973	**NELL1**	-1.06147	8.29E-05	0.014969
ENSG00000253598	**SLC10A5**	-1.03268	0.001644	0.047016
ENSG00000213918	**DNASE1**	-1.01007	4.42E-04	0.0292
ENSG00000169738	**DCXR**	-1.00106	7.23E-04	0.035209
ENSG00000164120	**HPGD**	-1.00002	0.001072	0.04088
ENSG00000146411	**SLC2A12**	-0.99292	3.19E-06	0.002935
ENSG00000123572	**NRK**	-0.97498	4.84E-04	0.029925
ENSG00000158865	**SLC5A11**	-0.96761	0.001344	0.044543
ENSG00000188488	**SERPINA5**	-0.95631	0.00135	0.044543
ENSG00000164089	**ETNPPL**	-0.94096	6.46E-05	0.013394
ENSG00000159208	**CIART**	-0.91506	0.001126	0.041319
ENSG00000115896	**PLCL1**	-0.83072	1.62E-04	0.019774
ENSG00000143257	**NR1I3**	-0.82029	0.001573	0.046235
ENSG00000118596	**SLC16A7**	-0.80558	0.001117	0.041319
ENSG00000066926	**FECH**	-0.79395	1.46E-05	0.007315
ENSG00000240303	**ACAD11**	-0.77193	0.001268	0.043693
ENSG00000150471	**ADGRL3**	-0.75157	5.85E-04	0.032195
ENSG00000106384	**MOGAT3**	-0.73672	2.89E-05	0.009308
ENSG00000134864	**GGACT**	-0.72921	6.53E-04	0.033262
ENSG00000130829	**DUSP9**	-0.71601	0.000806	0.036244
ENSG00000066185	**ZMYND12**	-0.65743	1.15E-04	0.017096
ENSG00000183715	**OPCML**	-0.6563	0.001043	0.040435
ENSG00000055163	**CYFIP2**	-0.65583	0.001031	0.040183
ENSG00000196482	**ESRRG**	-0.65537	0.001346	0.044543
ENSG00000248487	**ABHD14A**	-0.65185	4.41E-05	0.011184
ENSG00000163827	**LRRC2**	-0.64556	0.001702	0.047562
ENSG00000163145	**C1QTNF7**	-0.63128	0.001095	0.041027
ENSG00000165995	**CACNB2**	-0.61661	3.88E-05	0.010876
ENSG00000222726	**RNU2-7P**	-0.61175	7.24E-05	0.014017

ENSG00000050344	**NFE2L3**	0.600642	4.83E-05	0.011659
ENSG00000163739	**CXCL1**	0.604291	0.001056	0.040611
ENSG00000059804	**SLC2A3**	0.60466	0.001376	0.044543
ENSG00000125498	**KIR2DL1**	0.610991	7.39E-04	0.035209
ENSG00000132514	**CLEC10A**	0.611657	8.55E-04	0.037013
ENSG00000115935	**WIPF1**	0.615837	9.49E-04	0.038315
ENSG00000084070	**SMAP2**	0.617482	8.07E-04	0.036244
ENSG00000019582	**CD74**	0.618566	1.46E-04	0.019237
ENSG00000163840	**DTX3L**	0.620448	5.52E-04	0.03141
ENSG00000126882	**FAM78A**	0.621218	4.76E-04	0.029925
ENSG00000173821	**RNF213**	0.621884	6.26E-04	0.032356
ENSG00000204381	**LAYN**	0.624003	9.47E-04	0.038315
ENSG00000105329	**TGFB1**	0.624768	3.27E-04	0.024225
ENSG00000028137	**TNFRSF1B**	0.625874	4.43E-04	0.0292
ENSG00000149573	**MPZL2**	0.625921	1.92E-04	0.020767
ENSG00000102245	**CD40LG**	0.626306	8.01E-04	0.036244
ENSG00000130303	**BST2**	0.626796	0.001382	0.044543
ENSG00000132470	**ITGB4**	0.627779	2.48E-04	0.022636
ENSG00000142227	**EMP3**	0.634219	8.03E-04	0.036244
ENSG00000117152	**RGS4**	0.639962	6.11E-04	0.032195
ENSG00000172965	**MIR4435-2HG**	0.640109	3.23E-04	0.024171
ENSG00000147443	**DOK2**	0.640862	6.91E-04	0.034488
ENSG00000096433	**ITPR3**	0.642444	0.001264	0.043693
ENSG00000152229	**PSTPIP2**	0.643512	5.07E-04	0.030322
ENSG00000026508	**CD44**	0.643724	0.001267	0.043693
ENSG00000185885	**IFITM1**	0.645372	1.91E-04	0.020767
ENSG00000041353	**RAB27B**	0.645604	4.36E-04	0.0292
ENSG00000142512	**SIGLEC10**	0.649199	2.96E-05	0.009308
ENSG00000161921	**CXCL16**	0.653427	3.79E-04	0.026986
ENSG00000132334	**PTPRE**	0.656299	1.38E-04	0.018637
ENSG00000204397	**CARD16**	0.669665	0.00181	0.049258
ENSG00000133106	**EPSTI1**	0.673529	0.001196	0.042457
ENSG00000184922	**FMNL1**	0.680404	3.07E-04	0.024008
ENSG00000163814	**CDCP1**	0.680682	2.38E-05	0.009216
ENSG00000105383	**CD33**	0.682766	2.09E-04	0.021668
ENSG00000146192	**FGD2**	0.6832	4.32E-04	0.0292
ENSG00000197142	**ACSL5**	0.688155	4.50E-04	0.029221
ENSG00000090376	**IRAK3**	0.688245	0.001064	0.040812
ENSG00000174640	**SLCO2A1**	0.688293	1.26E-05	0.006883
ENSG00000162896	**PIGR**	0.690677	5.94E-04	0.032195
ENSG00000173706	**HEG1**	0.697256	1.49E-05	0.007315

ENSG00000159110	**IFNAR2**	0.697414	0.001416	0.045036
ENSG00000178860	**MSC**	0.703987	0.001575	0.046235
ENSG00000042980	**ADAM28**	0.709161	2.84E-04	0.023072
ENSG00000091490	**SEL1L3**	0.710355	0.001029	0.040183
ENSG00000163606	**CD200R1**	0.711873	0.001117	0.041319
ENSG00000155849	**ELMO1**	0.711903	2.76E-04	0.022979
ENSG00000151702	**FLI1**	0.718506	4.22E-04	0.028924
ENSG00000106560	**GIMAP2**	0.721162	0.001471	0.045785
ENSG00000137841	**PLCB2**	0.721463	0.001492	0.04602
ENSG00000115607	**IL18RAP**	0.722195	5.16E-04	0.030393
ENSG00000081320	**STK17B**	0.724236	0.001786	0.049116
ENSG00000198223	**CSF2RA**	0.726177	2.64E-04	0.022784
ENSG00000100351	**GRAP2**	0.72659	5.42E-04	0.03141
ENSG00000196610	**HLA-DQB2**	0.729401	0.001713	0.047672
ENSG00000206306	**HLA-DRB1**	0.742024	1.61E-04	0.019774
ENSG00000133816	**MICAL2**	0.744975	1.79E-04	0.020424
ENSG00000125538	**IL1B**	0.75109	0.001195	0.042457
ENSG00000183813	**CCR4**	0.751809	5.51E-04	0.03141
ENSG00000168961	**LGALS9**	0.752501	0.001164	0.041695
ENSG00000204287	**HLA-DRA**	0.753245	2.20E-04	0.022066
ENSG00000198821	**CD247**	0.754653	4.25E-04	0.028978
ENSG00000163563	**MNDA**	0.755867	9.10E-04	0.037486
ENSG00000142871	**CCN1**	0.759794	1.25E-04	0.017758
ENSG00000115523	**GNLY**	0.760631	6.33E-04	0.032612
ENSG00000132965	**ALOX5AP**	0.760736	1.36E-04	0.018515
ENSG00000117281	**CD160**	0.764164	0.001587	0.046235
ENSG00000101842	**VSIG1**	0.764626	3.21E-04	0.024171
ENSG00000180644	**PRF1**	0.769658	0.001823	0.049442
ENSG00000085265	**FCN1**	0.7705	0.001618	0.046727
ENSG00000118503	**TNFAIP3**	0.771029	0.001129	0.041319
ENSG00000100368	**CSF2RB**	0.771625	1.86E-04	0.020612
ENSG00000122223	**CD244**	0.773665	4.99E-04	0.030043
ENSG00000008517	**IL32**	0.77694	4.96E-04	0.030043
ENSG00000019991	**HGF**	0.77975	0.001581	0.046235
ENSG00000284567	**MIR223**	0.78047	2.84E-04	0.023072
ENSG00000005059	**MCUB**	0.787563	0.001757	0.048449
ENSG00000197471	**SPN**	0.793023	2.93E-05	0.009308
ENSG00000204252	**HLA-DOA**	0.806094	9.51E-04	0.038315
ENSG00000198959	**TGM2**	0.810457	7.15E-04	0.035209
ENSG00000205045	**SLFN12L**	0.81689	9.61E-04	0.038363
ENSG00000125245	**GPR18**	0.817215	0.001421	0.045036

ENSG00000111348	**ARHGDIB**	0.818001	1.68E-04	0.019821
ENSG00000266094	**RASSF5**	0.820213	0.001382	0.044543
ENSG00000165071	**TMEM71**	0.825573	4.38E-05	0.011184
ENSG00000144802	**NFKBIZ**	0.825804	0.001125	0.041319
ENSG00000185697	**MYBL1**	0.833267	4.09E-05	0.010876
ENSG00000101336	**HCK**	0.835629	3.49E-04	0.025577
ENSG00000133574	**GIMAP4**	0.837697	2.26E-04	0.022066
ENSG00000090339	**ICAM1**	0.840793	0.001456	0.045717
ENSG00000124491	**F13A1**	0.845172	5.68E-04	0.031801
ENSG00000135604	**STX11**	0.848144	7.75E-04	0.036015
ENSG00000132274	**TRIM22**	0.850257	2.83E-04	0.023072
ENSG00000172578	**KLHL6**	0.851218	2.75E-04	0.022979
ENSG00000254087	**LYN**	0.854501	8.95E-04	0.037397
ENSG00000145632	**PLK2**	0.857475	8.43E-04	0.036939
ENSG00000089327	**FXYD5**	0.85814	5.51E-05	0.012487
ENSG00000172236	**TPSAB1**	0.86063	1.95E-04	0.021013
ENSG00000168918	**INPP5D**	0.862177	3.76E-04	0.026986
ENSG00000172322	**CLEC12A**	0.863934	2.07E-04	0.021668
ENSG00000180353	**HCLS1**	0.867261	3.16E-04	0.02414
ENSG00000164691	**TAGAP**	0.86844	2.61E-05	0.009308
ENSG00000120280	**CXorf21**	0.870791	7.28E-04	0.035209
ENSG00000141968	**VAV1**	0.878006	2.53E-04	0.022636
ENSG00000131042	**LILRB2**	0.878767	0.001699	0.047562
ENSG00000138378	**STAT4**	0.887096	3.57E-04	0.025943
ENSG00000182578	**CSF1R**	0.888642	5.11E-04	0.030322
ENSG00000054219	**LY75**	0.889108	6.20E-04	0.032195
ENSG00000230708	**HLA-DPB1**	0.890903	1.42E-04	0.018948
ENSG00000204482	**LST1**	0.894485	9.19E-06	0.005636
ENSG00000010610	**CD4**	0.903951	0.001141	0.041389
ENSG00000130755	**GMFG**	0.904906	3.10E-04	0.024008
ENSG00000204359	**CFB**	0.906543	2.12E-04	0.02178
ENSG00000172236	**TPSAB1**	0.907105	1.65E-04	0.01981
ENSG00000115828	**QPCT**	0.907127	6.23E-05	0.013111
ENSG00000260314	**MRC1**	0.910657	0.001136	0.041319
ENSG00000260314	**MRC1**	0.910657	0.001136	0.041319
ENSG00000188042	**ARL4C**	0.911169	1.34E-04	0.018455
ENSG00000130775	**THEMIS2**	0.92713	2.32E-04	0.022066
ENSG00000174500	**GCSAM**	0.931149	2.73E-04	0.022979
ENSG00000206301	**HLA-DQA2**	0.934208	6.45E-04	0.032998
ENSG00000149534	**MS4A2**	0.935909	2.96E-04	0.023589
ENSG00000010671	**BTK**	0.945226	0.001346	0.044543

ENSG00000134539	**KLRD1**	0.947591	6.18E-04	0.032195
ENSG00000111913	**RIPOR2**	0.95112	2.33E-04	0.022066
ENSG00000110848	**CD69**	0.951876	7.77E-04	0.036015
ENSG00000106952	**TNFSF8**	0.959177	4.90E-04	0.029925
ENSG00000196664	**TLR7**	0.96303	0.001135	0.041319
ENSG00000135426	**TESPA1**	0.964969	2.70E-04	0.022979
ENSG00000065357	**DGKA**	0.967842	1.25E-04	0.017758
ENSG00000126860	**EVI2A**	0.968883	0.001167	0.041695
ENSG00000170542	**SERPINB9**	0.972216	7.94E-06	0.005584
ENSG00000116824	**CD2**	0.980459	0.001308	0.043967
ENSG00000111335	**OAS2**	0.981173	7.38E-04	0.035209
ENSG00000179639	**FCER1A**	0.985873	6.99E-04	0.034651
ENSG00000198574	**SH2D1B**	0.98624	3.80E-04	0.026986
ENSG00000007908	**SELE**	0.987998	0.001555	0.046181
ENSG00000072694	**FCGR2B**	1.00699	5.16E-04	0.030393
ENSG00000169413	**RNASE6**	1.010809	4.51E-04	0.029221
ENSG00000113532	**ST8SIA4**	1.019057	0.001526	0.046055
ENSG00000173578	**XCR1**	1.02103	0.000258	0.022646
ENSG00000110077	**MS4A6A**	1.026285	4.86E-04	0.029925
ENSG00000075884	**ARHGAP15**	1.026778	2.27E-04	0.022066
ENSG00000162692	**VCAM1**	1.027735	1.78E-04	0.020424
ENSG00000104974	**LILRA1**	1.036726	2.22E-04	0.022066
ENSG00000168421	**RHOH**	1.043168	4.00E-04	0.028118
ENSG00000116701	**NCF2**	1.046096	3.58E-04	0.025943
ENSG00000173372	**C1QA**	1.046398	0.001085	0.040974
ENSG00000023445	**BIRC3**	1.04726	6.62E-04	0.033625
ENSG00000173198	**CYSLTR1**	1.054175	5.00E-04	0.030043
ENSG00000043462	**LCP2**	1.056379	4.86E-04	0.029925
ENSG00000101347	**SAMHD1**	1.063944	5.83E-04	0.032195
ENSG00000070190	**DAPP1**	1.064237	1.21E-04	0.017599
ENSG00000187474	**FPR3**	1.072545	0.001514	0.046037
ENSG00000100385	**IL2RB**	1.081565	4.80E-04	0.029925
ENSG00000110324	**IL10RA**	1.084825	6.03E-04	0.032195
ENSG00000166927	**MS4A7**	1.092633	4.57E-04	0.029221
ENSG00000114013	**CD86**	1.094968	8.86E-04	0.037257
ENSG00000105851	**PIK3CG**	1.098284	7.44E-05	0.014039
ENSG00000196735	**HLA-DQA1**	1.10253	4.44E-04	0.0292
ENSG00000284353	**MIR142**	1.107554	0.001307	0.043967
ENSG00000113263	**ITK**	1.112563	5.52E-04	0.03141
ENSG00000143226	**FCGR2A**	1.114665	1.55E-04	0.019774
ENSG00000175857	**GAPT**	1.121213	2.58E-04	0.022646

ENSG00000101198	**NKAIN4**	1.126586	2.09E-04	0.021668
ENSG00000140030	**GPR65**	1.128013	2.37E-04	0.022077
ENSG00000122641	**INHBA**	1.130111	8.44E-05	0.014969
ENSG00000172673	**THEMIS**	1.135676	2.71E-04	0.022979
ENSG00000165168	**CYBB**	1.145901	0.001004	0.039435
ENSG00000134242	**PTPN22**	1.152943	0.001229	0.043198
ENSG00000173369	**C1QB**	1.154749	8.73E-04	0.037257
ENSG00000170006	**TMEM154**	1.169811	9.88E-05	0.015479
ENSG00000177272	**KCNA3**	1.171029	0.001151	0.041454
ENSG00000123338	**NCKAP1L**	1.184418	0.001377	0.044543
ENSG00000181631	**P2RY13**	1.186916	2.03E-06	0.002876
ENSG00000188404	**SELL**	1.193736	0.001497	0.04602
ENSG00000159189	**C1QC**	1.200005	7.53E-04	0.035647
ENSG00000140968	**IRF8**	1.201541	1.70E-04	0.019821
ENSG00000082074	**FYB1**	1.202938	5.52E-04	0.03141
ENSG00000111796	**KLRB1**	1.203754	7.63E-05	0.014232
ENSG00000115165	**CYTIP**	1.222124	4.48E-04	0.029221
ENSG00000005844	**ITGAL**	1.245586	0.001076	0.04088
ENSG00000166741	**NNMT**	1.255849	7.80E-04	0.036015
ENSG00000143110	**C1orf162**	1.259074	9.10E-05	0.015287
ENSG00000185811	**IKZF1**	1.261744	1.61E-04	0.019774
ENSG00000213088	**ACKR1**	1.264588	7.96E-06	0.005584
ENSG00000169442	**CD52**	1.284541	8.99E-04	0.037397
ENSG00000121807	**CCR2**	1.295125	5.08E-05	0.012062
ENSG00000158481	**CD1C**	1.310909	1.61E-04	0.019774
ENSG00000160593	**JAML**	1.311975	5.39E-05	0.012487
ENSG00000161929	**SCIMP**	1.330458	1.69E-04	0.019821
ENSG00000110876	**SELPLG**	1.340846	3.71E-05	0.010712
ENSG00000197629	**MPEG1**	1.356043	1.36E-05	0.007163
ENSG00000153283	**CD96**	1.363337	7.89E-04	0.036136
ENSG00000134061	**CD180**	1.363383	0.001428	0.045123
ENSG00000078589	**P2RY10**	1.366349	3.81E-04	0.026986
ENSG00000163519	**TRAT1**	1.376863	0.00154	0.046181
ENSG00000012779	**ALOX5**	1.393791	2.16E-06	0.002876
ENSG00000147168	**IL2RG**	1.411347	0.00127	0.043693
ENSG00000081237	**PTPRC**	1.423036	6.05E-04	0.032195
ENSG00000185862	**EVI2B**	1.477311	9.22E-05	0.015287
ENSG00000047457	**CP**	1.527655	9.04E-04	0.037397
ENSG00000203747	**FCGR3A**	1.53667	2.00E-04	0.021216
ENSG00000163751	**CPA3**	1.621545	1.80E-04	0.020424
ENSG00000168685	**IL7R**	1.691971	9.65E-04	0.038388

ENSG00000145287	**PLAC8**	1.73323	1.62E-04	0.019774
ENSG00000229092	**IGHV3-47**	1.734464	2.14E-06	0.002876
ENSG00000026751	**SLAMF7**	1.810957	7.35E-04	0.035209
ENSG00000254220	**IGKV2D-18**	1.906767	7.08E-05	0.014017
ENSG00000271130	**IGHV3OR16-8**	2.19727	2.46E-06	0.002876
ENSG00000259261	**IGHV4OR15-8**	2.408974	2.70E-05	0.009308
ENSG00000211957	**IGHV3-35**	2.467769	7.42E-07	0.002732
ENSG00000204780	**IGKV1OR9-1**	2.502481	1.98E-06	0.002876
ENSG00000241244	**IGKV1D-16**	2.51908	2.22E-05	0.009062
ENSG00000231292	**IGKV1OR2-108**	2.558745	1.15E-05	0.006607
ENSG00000253497	**IGKV1-13**	2.688964	4.64E-05	0.011485
ENSG00000243238	**IGKV2-30**	2.785034	2.33E-05	0.009216
ENSG00000242580	**IGKV1D-43**	2.798225	8.60E-05	0.015081
ENSG00000211955	**IGHV3-33**	2.827125	2.77E-06	0.002876
ENSG00000241566	**IGKV2D-24**	2.836495	4.68E-05	0.011485
ENSG00000224650	**IGHV3-74**	2.85097	2.65E-06	0.002876
ENSG00000271620	**IGHV3OR16-7**	2.875485	2.23E-06	0.002876
ENSG00000224607	**IGKV1D-27**	2.964389	8.80E-04	0.037257
ENSG00000278857	**IGKV1D-12**	3.000936	3.04E-04	0.023946
ENSG00000239975	**IGKV1D-33**	3.043776	1.17E-05	0.006607
ENSG00000211938	**IGHV3-7**	3.096687	4.58E-07	0.002732
ENSG00000270505	**IGHV1OR15-1**	3.112992	1.29E-04	0.018145
ENSG00000233999	**IGKV3OR2-268**	3.241442	2.95E-05	0.009308
ENSG00000224373	**IGHV4-59**	3.29686	1.88E-05	0.008093
ENSG00000211632	**IGKV3D-11**	3.359391	5.88E-05	0.012557
ENSG00000211623	**IGKV2D-26**	3.517572	1.92E-05	0.008093
ENSG00000132465	**JCHAIN**	3.677296	6.19E-06	0.004797
